# Mathematical Model of the Impact of a Nonantibiotic Treatment for *Clostridium difficile* on the Endemic Prevalence of Vancomycin-Resistant Enterococci in a Hospital Setting

**DOI:** 10.1155/2012/605861

**Published:** 2012-01-15

**Authors:** Daniel T. Grima, Glenn F. Webb, Erika M. C. D'Agata

**Affiliations:** ^1^Cornerstone Research Group Inc., 204-3228 South Service Road, Burlington, ON, Canada L7N 3H8; ^2^Mathematics Department, Vanderbilt University, Nashville, TN, 37240, USA; ^3^Division of Infectious Disease, Beth Israel Deaconess Medical Center, Harvard Medical School, Lowry Building Suite 6A, 100 Francis Street, Boston, MA, 02215, USA

## Abstract

*Introduction*. *Clostridium difficile*-associated disease (CDAD) is treated using antibiotics, which often leads to the emergence of antibiotic-resistant bacteria such as vancomycin-resistant enterococci (VRE). This study estimated the impact of a non antibiotic treatment for CDAD on VRE prevalence. *Methods*. A previously published model describing the impact of in-hospital antibiotic use on VRE prevalence was adapted to include CDAD treatment. Simulations compared the prevalence of VRE when nonantibiotic versus antibiotic therapy was used. *Results*. Nonantibiotic treatment in 50% of CDAD patients resulted in an 18% relative reduction in the prevalence of VRE colonization compared with antibiotic use only. Sensitivity analysis found the model to be most sensitive to rates of antibiotic initiation and discontinuation, prevalence of VRE in admitted patients, length of stay of colonized patients, probability of CDAD acquisition, and hand-washing compliance. *Conclusion*. Nonantibiotic treatment of patients hospitalized with CDAD may significantly reduce the incidence of VRE colonization.

## 1. Introduction

The bacterium* Clostridium difficile* is the most common cause of hospital-acquired diarrhea, accounting for up to 25% of all cases of antibiotic-associated diarrhea [1, 2]. Rates of colonization of inpatients with *C. difficile* may be as high as 50% for patients hospitalized for more than four weeks [3]. The incidence of *C. difficile*-associated disease (CDAD) has been growing on the basis of data from the National Nosocomial Infections Surveillance system [3]. Importantly, outbreaks of severe CDAD cases have occurred in several hospitals because of the emergence of a new *C*. *difficile* strain with increased virulence and antibiotic resistance [4, 5]. This more virulent bacterial strain results in more admissions to the intensive care unit, colectomies, and death than other strains [4, 5]. A recent study reported that in the United States, the incidence of adult CDAD hospitalizations doubled from 5.5 cases per 10,000 population in 2000 to 11.2 per 10,000 cases in 2005, and the age-adjusted CDAD-related case-fatality rate rose from 1.2% in 2000 to 2.2% in 2004 [6]. 

Current treatment of CDAD involves antibiotic therapy with either metronidazole or vancomycin. However, there has been a growing interest in the development of nonantibiotic therapies for CDAD and other nosocomial infections in order to reduce antibiotic use in hospitals, where the goal is to limit the emergence and spread of antibiotic-resistant bacteria such as vancomycin-resistant enterococci (VRE) [7]. The use of such antibiotics can eradicate antibiotic-susceptible bacterial strains in the gastrointestinal flora, thereby allowing the overgrowth of subpopulations of antibiotic-resistant bacteria [8, 9]. In the case of *C*. *difficile *treatment, both metronidazole and vancomycin are associated with the promotion of VRE. This overgrowth is evidenced by the cocolonization of *C*. *difficile* and VRE that occurs in 20% to 34% of patients, which may reflect *C*. *difficile*-directed antibiotic exposure as well as other common risk factors for these pathogens [10–12].

It has been suggested that nonantibiotic treatment of CDAD may offer an opportunity to reduce antibiotic use in hospitals and could ultimately decrease the prevalence and burden of VRE. For example, although ultimately unsuccessful, the enterotoxin-binding polymer tolevamer (Genzyme, Inc.) was investigated for the treatment of CDAD in 2008 [13, 14]. More recently, a number of small studies have reported on the successful use of fecal donor instillation therapy (FDIT) for the treatment of patients with CDAD [15–17]. Used for decades in some hospitals, only recently has the effectiveness of this therapy been documented in the literature. In a retrospective study of 40 patients with recurrent CDAD, 83% of patients were successfully treated with FDIT, having met the study's endpoint of no further hospital contact due to CDAD symptoms within 80 days of therapy. While in the study's protocol patients were treated with metronidazole or vancomycin until reduction of symptoms, all antimicrobial therapy was discontinued on the evening prior to donor stool transplantation [18].

The objective of this study was to estimate the potential impact of a nonantibiotic treatment for CDAD on the prevalence of VRE within a hospital setting. A mathematical model was used to estimate and compare the prevalence of VRE with current antibiotic therapies for CDAD versus nonantibiotic therapies such as FDIT.

## 2. Methods

**Figure 1:**
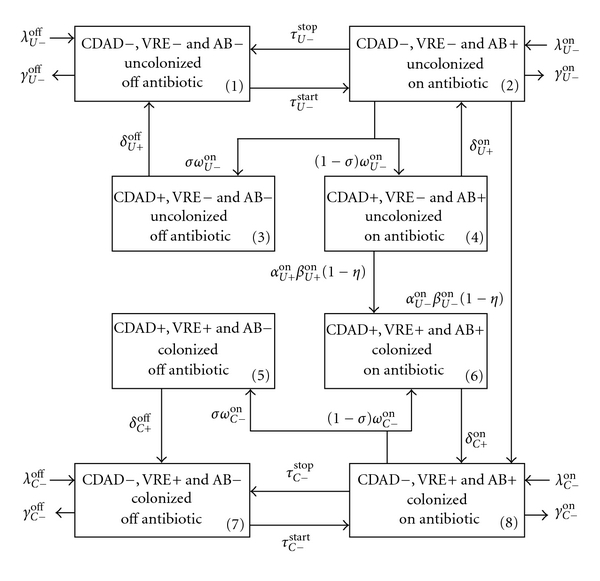
Schematic diagram of the patient compartments. CDAD = *clostridium difficile*-associated disease; CDAD+ = *clostridium difficile*-associated disease positive; CDAD− = *clostridium difficile*-associated disease negative; VRE = vancomycin-resistant enterococci; VRE+ = vancomycin-resistant enterococci colonized; VRE− = vancomycin-resistant enterococci not colonized; AB− = off antibiotics; AB+ = on antibiotics; *λ*_*U*−_^*off*⁡^ = CDAD−, uncolonized, off antibiotic: admission rate; *γ*_*U*−_^*off*⁡^= CDAD−, uncolonized, off antibiotic: average length of stay; *λ*_*U*−_^*on*⁡^= CDAD−, uncolonized, on antibiotic: admission rate; *γ*_*U*−_^*on*⁡^= CDAD−, uncolonized, on antibiotic: average length of stay; *τ*_*U*−_^stop^= CDAD−, uncolonized, on antibiotic: stop antibiotic; *τ*_*U*−_^start^= CDAD−, uncolonized, off antibiotic: start antibiotic; *δ*_*U*+_^*off*⁡^= CDAD+, uncolonized, off antibiotic: transit to CDAD−; *σ* = fraction of AB patients that transit to CDAD+ and stop AB; *ω*_*U*−_^*on*⁡^= CDAD−, uncolonized, on antibiotic: transit to CDAD+; *δ*_*U*+_^*on*⁡^ = CDAD+, uncolonized, on antibiotic: transit to CDAD−; *α*_*U*+_^*on*⁡^= average number of contacts between uncolonized CDAD− patients on antibiotic and HCW; *β*_*U*+_^*on*⁡^= probability of colonization per contact between uncolonized CDAD+ patient on antibiotic and contaminated HCW; *α*_*U*−_^*on*⁡^= average number of contacts between uncolonized CDAD+ patients on antibiotic and HCW; *β*_*U*−_^*on*⁡^= probability of colonization per contact between uncolonized CDAD− patient on antibiotic and contaminated HCW; *η* = hand washing compliance factor between 0 and 1; *δ*_*C*+_^*off*⁡^= CDAD+, colonized, off antibiotic: transit to CDAD−; *ω*_*C*−_^*on*⁡^= CDAD−, colonized, on antibiotic: transit to CDAD+; *δ*_*C*+_^*on*⁡^ = CDAD+, colonized, on antibiotic: transit to CDAD−; *λ*_*C*−_^*off*⁡^= CDAD−, colonized, off antibiotic: admission rate; *τ*_*C*−_^stop^= CDAD−, colonized, on antibiotic: stop antibiotic; *λ*_*C*−_^*on*⁡^= CDAD−, colonized, on antibiotic: admission rate; *τ*_*C*−_^start^= CDAD−, colonized, off antibiotic: start antibiotic; *γ*_*C*−_^*on*⁡^= CDAD−, colonized, on antibiotic: average length of stay; *γ*_*C*−_^*off*⁡^= CDAD−, colonized, off antibiotic: average length of stay.

The current model (Appendix) extends a previously published model by D'Agata and colleagues (2005) that described the impact of antibiotic use on VRE prevalence within a hospital setting [19]. The model considers the admission of patients with and without VRE to a hospital and their consequent risks of CDAD and VRE acquisition. Since VRE is predominantly spread from patient-to-patient via the contaminated hands or clothes of health care workers (HCWs), the complex interactions between patients and HCWs were also incorporated into the model. The model describes the transmission dynamics of VRE in a 400-bed hospital. The original model compartmentalized patients into those receiving (right side of Figure 1) and not receiving (left side of Figure 1) antibiotics, and those who were VRE-colonized (bottom boxes in Figure 1) and those who were not (top boxes in Figure 1). The original model was then extended to include CDAD status. Each of the four original boxes was divided into an outer and inner box, and CDAD positive (CDAD+) patients were included in the inner boxes 3, 4, 5, and 6. These boxes included CDAD patients who were not receiving antibiotics and were either uncolonized (Box 3) or colonized (Box 5) with VRE as well as individuals who were receiving antibiotics and were either uncolonized (Box 4) or colonized (Box 6) with VRE.

**Figure 2:**
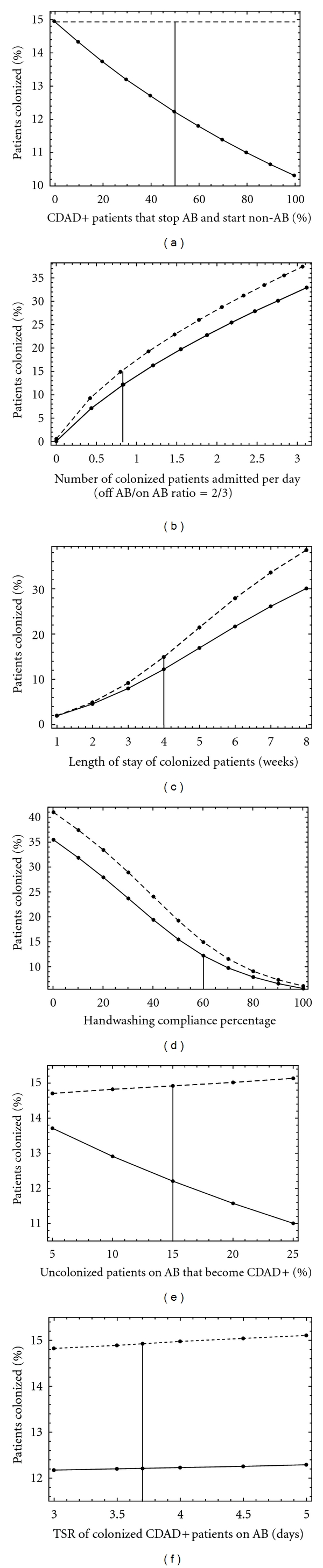
Sensitivity analyses of the model parameters. Dashed lines: Scenario 1 (use of AB only); Solid lines: Scenario 2 (use of non-anti-microbial); Vertical line: baseline values; (a) Percentage of CDAD+ patients that stop AB and start non-AB (baseline = 50%, *σ* = 0.5); (b) Number of colonized patients admitted per day in the ratio *λ*_*C*−_^*off*⁡^/*λ*_*C*−_^*on*⁡^ = 2/3 (baseline *λ*_*C*−_^*off*⁡^ = 0.33, *λ*_*C*−_^*on*⁡^ = 0.5); (c) Length of stay of colonized patients in weeks (baseline = 4 weeks, = *γ*_*C*−_^*off*⁡^/*γ*_*C*−_^*on*⁡^= 1/28); (d) Hand-washing compliance percentage (baseline = 60%, *η* = 0.6); (e) Percentage of uncolonized and colonized patients on AB (held equal) that become CDAD+ (baseline = 15%, *ω*_*U*−_^*on*⁡^ = *ω*_*C*−_^*on*⁡^ = −log (0.9893)); (f) Time to symptoms resolution (TSR) of colonized CDAD+ patients on AB in days (baseline = 3.7 days, *δ*_*C*+_^*on*⁡^ = −log (0.73)); AB = antibiotic; CDAD = *clostridium difficile*-associated disease; non-AB = nonantibiotic; *λ*_*C*−_^*off*⁡^ = CDAD−, colonized, off antibiotic: admission rate; *γ*_*C*−_^*on*⁡^ = CDAD−, colonized, on antibiotic: average length of stay; *ω*_*U*−_^*on*⁡^ = CDAD−, uncolonized, on antibiotic: transit to CDAD+; *ω*_*C*−_^*on*⁡^ = CDAD−, colonized, on antibiotic: transit to CDAD+; *γ*_*C*−_^*off*⁡^ = CDAD−, colonized, off antibiotic: average length of stay; *λ*_*C*−_^*on*⁡^ = CDAD−, colonized, on antibiotic: admission rate.

All model parameter values are provided in Table 1. Except where noted below, the same values from D'Agata and colleagues' model were used. These values were originally obtained from pharmacy records and an observational HCW study at Beth Israel Deaconess Medical Center [19]. The spread of VRE between patients and associated movement between compartments occurred because of HCWs whose hands were contaminated with VRE after contact with a colonized patient (Figure 2). The interaction between patients and HCWs included the number of contacts with a patient during routine patient care and compliance with hand hygiene (Table 1). The latter was an important inclusion, since hand hygiene will remove VRE from HCWs' hands and thereby prevent transmission of VRE to patients. It was assumed that patients who were colonized with VRE were on contact precautions and therefore less likely to contaminate HCWs. The implementation of these precautions is standard practice in hospital settings and requires HCWs to don gloves and gowns prior to entering the room of a VRE-colonized patient in order to prevent HCW contamination [20].

The movement between compartments was also influenced by the proportion of patients who were started on antibiotics or in whom antibiotics were discontinued. It was assumed that patients receiving antibiotics were more likely to contaminate an HCW, since studies have documented that antibiotic exposure results in increased VRE stool densities and a greater likelihood of skin contamination (Table 1) [9]. Although contamination of HCWs' clothes and inanimate surfaces contribute to VRE dissemination, these factors were omitted in order to simplify the model [21].

The probability of colonization per contact between uncolonized CDAD patients on antibiotics and contaminated HCW (0.16) was based on an assumption that contact precautions for CDAD+ patients would decrease the probability of contamination by 60% compared to patients without CDAD [22]. These values were obtained from studies focusing on *Acinetobacter* spp. The daily probability of patients on antibiotics to transit to CDAD+ (15/14 = 1.07%) was based on an overall risk of CDAD infection for antibiotic-treated patients of 15% and an average length of stay of 14 days, as per the original model [19]. The daily probability of CDAD resolution of 27.03% = 100 (3 × 9 + 10 × 0.1) was based on the assumption that 90% of CDAD cases resolve within 3 days and 10% require 10 days [23].

The model was run until it reached a steady state, and the total numbers of patients in each state was compared in two scenarios: (1) all patients received antibiotics for treatment of CDAD in hospital and (2) a subgroup of CDAD patients received a nonantibiotic treatment for CDAD with equal efficacy to antibiotic therapy. First, to simulate current care for CDAD with antibiotic therapy and the lack of a nonantibiotic treatment option, the transition probabilities for entry into boxes 3 and 5 were set to zero. Second, to simulate the impact of avoiding antibiotic exposure with the use of a nonantibiotic therapy for CDAD, 50% of patients acquiring CDAD were assumed to discontinue antibiotics and move to boxes 3 and 5.

## 3. Sensitivity Analyses

Univariate sensitivity analyses were conducted for percent of CDAD+ patients that stop antibiotics and start a nonantibiotic treatment, number of colonized patients admitted per day, length of stay of colonized patients, percentage of hand-washing compliance, percentage of uncolonized patients on antibiotics that become CDAD+, and time to symptoms resolution of colonized CDAD+ patients on antibiotics.

## 4. Results

The distributions of patients in each steady state under both scenarios are summarized in Table 2. Under the first scenario, where antibiotics were used for all CDAD+ patients (*σ* = 0.0), of the approximately 400 patients in the steady state, 60 (15.0%) were colonized with VRE, 194 (48.5%) were on antibiotics, and 6 (1.5%) were CDAD+, all of whom were on antibiotics (Table 2). Under the second scenario, where 50% of CDAD+ patients discontinued antibiotics in favor of a nonantibiotic treatment (*σ* = 0.5), VRE colonization was reduced. In this scenario, the total number of patients on antibiotics declined to 185 (46.3%) and the number of CDAD+ patients receiving antibiotics declined to 3 (0.75%). The total number of patients who were VRE-colonized at any given time declined from 60 to 49 (from 15.0% down to 12.25%), an absolute decline of 2.75% and a relative decline of 18%. Assuming a 400-bed hospital and 25 patients per bed a year, this outcome translates into 275 avoided cases of VRE colonization per year.

Results of the sensitivity analyses are presented in Figure 2. In all analyses, the nonantibiotic use scenario was associated with a lower prevalence of VRE colonization. In the nonantibiotic scenario, the percentage of VRE-colonized patients decreased notably if the percentage of CDAD+ patients that stop antibiotics and start a nonantibiotic regiment increased (Figure 2(a)), the hand-washing compliance percentage increased (Figure 2(d)), or the percentage of uncolonized patients on antibiotics that become CDAD+ increased (Figure 2(e)). With both antibiotic and nonantibiotic therapies, the percentage of VRE-colonized patients increased notably when the number of colonized patients admitted per day increased (Figure 2(b)) or the length of stay of colonized patients increased (Figure 2(c)).

## 5. Discussion

The association between antibiotic use in hospitals and the colonization of patients with antibiotic-resistant bacteria such as VRE is well documented [24]. This finding has spurred research into nonantibiotic options for the treatment of nosocomial infections such as CDAD. For example, the anionic polymer tolevamer (Genzyme, Inc.) was developed to neutralize clostridial toxins without adversely affecting the normal flora of the gut [13, 14]. Results from a phase III trial revealed that the recurrence rate of CDAD was reduced significantly when compared with vancomycin and metronidazole; however, the study failed to meet its primary endpoint due to the high dropout rate associated with tolevamer [25]. As the development of the therapy was subsequently halted, its impact on the prevalence of VRE is unknown.

More recent publications have reported on the safety and effectiveness of FDIT for the treatment of CDAD. This therapy involves the introduction of a stool transplant into the duodenum or colon via a gastroscope or colonoscope, respectively, to repopulate the microflora of the bowel [15, 16]. Although used for decades, particularly in Scandinavian countries, the clinical data describing FDIT are limited; still, available reports suggest that the treatment is safe and effective even in patients with refractory CDAD [15–17]. A recent review of 16 publications concluded that 91% of all reported patients with recurrent *C. difficile* infection who were treated with FDIT were cured after one or more infusions [26]. The impact of FDIT on the prevalence of VRE, however, remains uncertain.

The availability of a nonantibiotic treatment for CDAD raises interesting questions regarding its possible impact on the prevalence of VRE compared with conventional antibiotic therapy. Our study adapted an existing model of VRE prevalence within a hospital setting to study the possible effect of the introduction of a nonantibiotic treatment for CDAD. The study found that treatment of 50% of patients with CDAD with a nonantibiotic regimen resulted in an approximately 2.75% absolute reduction (18% relative reduction) in VRE-colonized patients compared with a scenario, whereby all CDAD patients received antibiotics.

This reduction in VRE prevalence represents a meaningful impact within a hospital, given the costs associated with the management of VRE-colonized patients and potential clinical impact of VRE infections. For a 400-bed hospital, the analysis estimated a reduction of 275 cases of VRE colonization. On the basis of an average cost of contact precautions of USD $2,694.01 (CDN $3,191.83 at exchange of 0.844034), this would result in cost savings of approximately USD $740,800 a year [27]. A small reduction in VRE prevalence may also have a substantial impact on the clinical outcome and costs of VRE infections. VRE infection has been observed to occur in as many as 11.1% of VRE-colonized patients [12]. The cost to treat a patient with a VRE infection has been reported as approximately USD $13,000 [28]. The mortality rate for a VRE infection has been estimated as 39% to 49% [29]. On the basis of the current model, an 18% reduction in VRE prevalence could avert as many as 30.5 cases of VRE infection for a 400-bed hospital. This reduction in VRE infections could lead to a cost savings of USD $396,500 per year and could prevent up to 15 VRE-associated deaths annually.

Note that the current study excludes other types of antibiotic-resistant bacteria such as methicillin-resistant *Staphylococcus aureus*, which would also be affected by reduced use of antibiotics within a hospital. As such, it is likely that the study underestimates the extent of the impact of a nonantibiotic treatment for CDAD.

The study shares limitations that are common to all mathematical models in that it requires the use of data from multiple sources to model processes that have not been fully observed. The validity of such a model comes from the use of realistic assumptions and clinical review. In this case, the study was derived from a published model that was based on patterns of VRE transmission that have been widely studied and baseline parameters that were obtained from actual hospital data [19]. Given the variability that may exist for some of the model parameters, extensive sensitivity analyses were conducted. These sensitivity analyses revealed that the following variables had the most influence on the prevalence of VRE: the rate of antibiotic initiation or discontinuation in CDAD negative and positive patients, the length of stay of VRE-colonized patients, hand-washing compliance, and the number of VRE-colonized patients admitted per day. All of these variables would be expected to differ among hospitals given local infection control policies and the endemic rates of VRE colonization in the community.

## 6. Conclusions

Use of a nonantibiotic treatment in hospital patients with CDAD may significantly reduce the colonization of patients with VRE and the associated burden of disease.

## Figures and Tables

**Table 1:** Parameters used in modeling the effect of antibiotic versus nonantibiotic treatment of CDAD on VRE colonization.

Parameter	Patients	Value
*Np*	number of patients in the hospital	400
*ρ*	ratio of patients to HCW	4
*λ*_*U*−_^*off*⁡^	CDAD−, uncolonized, off antibiotic: admission rate	50.1 per day
*γ*_*U*−_^*off*⁡^	CDAD−, uncolonized, off antibiotic: average length of stay	5 days
*λ*_*U*−_^*on*⁡^	CDAD−, uncolonized, on antibiotic: admission rate	2.505 per day
*γ*_*U*−_^*on*⁡^	CDAD−, uncolonized, on antibiotic: average length of stay	14 days
*λ*_*C*−_^*off*⁡^	CDAD−, colonized, off antibiotic: admission rate	0.334 per day
*γ*_*C*−_^*off*⁡^	CDAD−, colonized, off antibiotic: average length of stay	28 days
*λ*_*C*−_^*on*⁡^	CDAD−, colonized, on antibiotic: admission rate	0.501 per day
*γ*_*C*−_^*on*⁡^	CDAD−, colonized, on antibiotic: average length of stay	28 days
*τ*_*U*−_^stop^	CDAD−, uncolonized, on antibiotic: stop antibiotic	15% per day
*τ*_*U*−_^start^	CDAD−, uncolonized, off antibiotic: start antibiotic	15% per day
*τ*_*C*−_^stop^	CDAD−, colonized, on antibiotic: stop antibiotic	4% per day
*τ*_*C*−_^start^	CDAD−, colonized, off antibiotic: start antibiotic	16% per day
*σ*	fraction of CDAD+, on antibiotic: stop antibiotic	0.5
*α*_*U*+_^*on*⁡^	average number of contacts between uncolonized	8 per day
	CDAD− patients on antibiotic and HCW	
*α*_*U*−_^*on*⁡^	average number of contacts between uncolonized	8 per day
	CDAD+ patients on antibiotic and HCW	
*β*_*U*+_^*on*⁡^	probability of colonization per contact between uncolonized	0.024
	CDAD+ patient on antibiotic and contaminated HCW	
*β*_*U*−_^*on*⁡^	probability of colonization per contact between uncolonized	0.06
	CDAD− patient on antibiotic and contaminated HCW	
*ω*_*U*−_^*on*⁡^	CDAD−, uncolonized, on antibiotic: transit to CDAD+	1.07% per day
*ω*_*C*−_^*on*⁡^	CDAD−, colonized, on antibiotic: transit to CDAD+	1.07% per day
*δ*_*C*+_^*on*⁡^	CDAD+, colonized, on antibiotic: transit to CDAD−	27% per day
*δ*_*C*+_^*off*⁡^	CDAD+, colonized, off antibiotic: transit to CDAD−	27% per day
*δ*_*U*+_^*on*⁡^	CDAD+, uncolonized, on antibiotic: transit to CDAD−	27% per day
*δ*_*U*+_^*off*⁡^	CDAD+, uncolonized, off antibiotic: transit to CDAD−	27% per day
*σ*	fraction of AB patients that transit to CDAD+ and stop AB	0.0 or 0.5

Parameter	Health care workers	Value

*α*_*C*+_	average number of contacts between HCW and colonized CDAD+ patients	10 per day
*α*_*C*−_	average number of contacts between HCW and colonized CDAD+ patients	10 per day
*κ*_−_^*on*⁡^	probability of contamination per contact between uncontaminated	0.4
	HCW and colonized CDAD− patient on antibiotic	
*κ*_−_^*off*⁡^	probability of contamination per contact between uncontaminated	0.4
	HCW and colonized CDAD− patient off antibiotic	
*κ*_+_^*on*⁡^	probability of contamination per contact between uncontaminated	0.16
	HCW and colonized CDAD+ patient on antibiotic	
*κ*_+_^*off*⁡^	probability of contamination per contact between uncontaminated	0.16
	HCW and colonized CDAD+ patient off antibiotic	
*μ*	average duration of HCW contamination	48 minutes
*η*	hand-washing compliance factor between 0 and 1	0.6

CDAD = *clostridium difficile*-associated disease; VRE = vancomycin-resistant enterococci; HCW = health care worker; CDAD+ = *clostridium difficile*-associated disease positive; CDAD− = *clostridium difficile*-associated disease negative; AB = antibiotics.

**Table 2:** Distribution of patients and health care workers with or without VRE colonization on the basis of antibiotic versus nonantibiotic treatment of CDAD.

Patient colonization/HCW contamination status	AB status	Model with AB only	Model with AB and non-AB	Change with non-AB	Difference with non-AB
Patient Steady State Values					

uncolonized CDAD− patient	off	195.176	202.555	increases	7.390
uncolonized CDAD− patient	on	140.383	143.553	increases	3.170
colonized CDAD− patient	off	10.7197	9.0598	decreases	−1.6599
colonized CDAD− patient	on	47.3343	38.4389	decreases	−8.8954
uncolonized CDAD+ patient	on	4.7467	2.4307	decreases	−2.3160
colonized CDAD+ patient	on	1.6721	0.6798	decreases	−0.9923
uncolonized CDAD+ patient	off	0.0	2.4535	increases	2.4535
colonized CDAD+ patient	off	0.0	0.6570	increases	0.6570

Patient Totals at Steady State					

total patients		400.03	399.828		
Total VRE-colonized patients		59.7361	48.83555	decreases	−10.8905
total CDAD+ patients		6.4188	6.2210	decreases	−0.1978
total CDAD+ patients on AB		6.4188	3.1105	decreases	−3.3083
total patients on AB		194.136	185.102	decreases	−9.034

Percentage of Patients Colonized at Steady State					

% of all patients colonized		14.930%	12.214%	decreases	−2.716%
% CDAD− patients colonized		14.749%	12.068%	increases	−2.681%
% CDAD+ patients colonized		26.050%	21.488%	decreases	−4.562%

HCW Steady State Values					

uncontaminated HCW		95.3347	96.1513	increases	0.8166
contaminated HCW		4.6653	3.8487	decreases	−0.8166

CDAD = *Clostridium difficile*-associated disease; VRE = vancomycin-resistant enterococci; HCW = health care worker; CDAD+ = *Clostridium difficile*-associated disease positive; CDAD− = *clostridium difficile*-associated disease negative; AB = antibiotic.

## Appendix

The CDAD epidemic within the hospital setting is described mathematically by a system of nonlinear ordinary differential equations. The populations under consideration are as follows: uncolonized CDAD− patients off antibiotics (*P*_*U*−_^*off*⁡^), uncolonized CDAD− patients on antibiotics (*P*_*U*−_^*on*⁡^), colonized CDAD− patients off antibiotics (*P*_*C*−_^*off*⁡^), colonized CDAD− patients on antibiotics (*P*_*C*−_^*on*⁡^), uncolonized CDAD^+^ patients off antibiotics (*P*_*U*+_^*off*⁡^), uncolonized CDAD^+^ patients on antibiotics (*P*_*U*+_^*on*⁡^), colonized CDAD^+^ patients off antibiotics (*P*_*C*+_^*off*⁡^), colonized CDAD^+^ patients on antibiotics (*P*_*C*+_^*on*⁡^), uncontaminated health care workers (*H*_*U*_), and contaminated health care workers (*H*_*C*_). Each population is a function of time (*t*) in days, and each population has initial conditions prescribed at time 0. The rates of change of these populations are governed by

(A.1) $$\begin{array}{cc} \frac{dP_{U-}^{\mathrm{off}}}{dt} & =\lambda_{U-}^{\mathrm{off}}-\tau_{U-}^{\text{start}}P_{U-}^{\mathrm{off}}+\tau_{U-}^{\text{stop}}P_{U-}^{\mathrm{on}}-\gamma_{U-}^{\mathrm{off}}P_{U-}^{\mathrm{off}}+\delta_{U+}^{\mathrm{off}}P_{U+}^{\mathrm{off}},\\ \frac{dP_{U-}^{\mathrm{on}}}{dt} & =\lambda_{U-}^{\mathrm{on}}-\tau_{U-}^{\text{stop}}P_{U-}^{\mathrm{on}}+\tau_{U-}^{\text{start}}P_{U-}^{\mathrm{off}}-\omega_{U-}^{\mathrm{on}}P_{U-}^{\mathrm{on}}\\ & \;-\alpha_{U-}^{\mathrm{on}}\beta_{U-}^{\mathrm{on}}\left(1-\eta \right)\frac{H_{C}}{Nh}P_{U-}^{\mathrm{on}}+\delta_{U+}^{\mathrm{on}}P_{U+}^{\mathrm{on}}-\gamma_{U-}^{\mathrm{on}}P_{U-}^{\mathrm{on}},\\ \frac{dP_{U+}^{\mathrm{off}}}{dt} & =\sigma \omega_{U-}^{\mathrm{on}}P_{U-}^{\mathrm{on}}-\delta_{U+}^{\mathrm{off}}P_{U+}^{\mathrm{off}},\\ \frac{dP_{U+}^{\mathrm{on}}}{dt} & =\left(1-\sigma \right)\omega_{U-}^{\mathrm{on}}P_{U-}^{\mathrm{on}}-\delta_{U+}^{\mathrm{on}}P_{U+}^{\mathrm{on}}\\ & \;-\alpha_{U+}^{\mathrm{on}}\beta_{U+}^{\mathrm{on}}\left(1-\eta \right)\frac{H_{C}}{Nh}P_{U+}^{\mathrm{on}},\\ \frac{dP_{C+}^{\mathrm{off}}}{dt} & =\sigma \omega_{C-}^{\mathrm{on}}P_{C-}^{\mathrm{on}}-\delta_{C+}^{\mathrm{off}}P_{C+}^{\mathrm{off}},\\ \frac{dP_{C+}^{\mathrm{on}}}{dt} & =\left(1-\sigma \right)\omega_{C-}^{\mathrm{on}}P_{C-}^{\mathrm{on}}-\delta_{C+}^{\mathrm{on}}P_{C+}^{\mathrm{on}}\\ & \;+\alpha_{U+}^{\mathrm{on}}\beta_{U+}^{\mathrm{on}}\left(1-\eta \right)\frac{H_{C}}{Nh}P_{U+}^{\mathrm{on}},\\ \frac{dP_{C-}^{\mathrm{off}}}{dt} & =\lambda_{C-}^{\mathrm{off}}-\tau_{C-}^{\text{start}}P_{C-}^{\mathrm{off}}+\tau_{C-}^{\text{stop}}P_{C-}^{\mathrm{on}}\\ & \;-\gamma_{C-}^{\mathrm{off}}P_{C-}^{\mathrm{off}}+\delta_{C+}^{\mathrm{off}}P_{C+}^{\mathrm{off}},\\ \frac{dP_{C-}^{\mathrm{on}}}{dt} & =\lambda_{C-}^{\mathrm{on}}-\tau_{C-}^{\text{stop}}P_{C-}^{\mathrm{on}}+\tau_{C-}^{\text{start}}P_{C-}^{\mathrm{off}}-\omega_{C-}^{\mathrm{on}}P_{C-}^{\mathrm{on}}\\ & \;+\alpha_{U-}^{\mathrm{on}}\beta_{\text{U}-}^{\mathrm{on}}\left(1-\eta \right)\frac{H_{C}}{Nh}P_{U-}^{\mathrm{on}}+\delta_{C+}^{\mathrm{on}}P_{C+}^{\mathrm{on}}-\gamma_{C-}^{\mathrm{on}}P_{C-}^{\mathrm{on}},\\ \frac{dH_{U}}{dt} & =-[\alpha_{C-}\kappa_{-}^{\mathrm{off}}\left(\frac{P_{C-}^{\mathrm{off}}}{Np}\right)+\alpha_{C-}\kappa_{-}^{\mathrm{on}}\left(\frac{P_{C-}^{\mathrm{on}}}{Np}\right)\\ & \;\;\;+\alpha_{C+}\kappa_{+}^{\mathrm{on}}\left(\frac{P_{C+}^{\mathrm{on}}}{Np}\right)+\alpha_{C+}\kappa_{+}^{\mathrm{off}}\left(\frac{P_{C+}^{\mathrm{off}}}{Np}\right)]\\ & \;\times \rho H_{U}+\mu H_{C},\\ \frac{dH_{C}}{dt} & =[\alpha_{C-}\kappa_{-}^{\mathrm{off}}\left(\frac{P_{C-}^{\mathrm{off}}}{Np}\right)+\alpha_{C-}\kappa_{-}^{\mathrm{on}}\left(\frac{P_{C-}^{\mathrm{on}}}{Np}\right)\\ & \;\;+\alpha_{C+}\kappa_{+}^{\mathrm{on}}\left(\frac{P_{C+}^{\mathrm{on}}}{Np}\right)+\alpha_{C+}\kappa_{+}^{\mathrm{off}}\left(\frac{P_{C+}^{\mathrm{off}}}{Np}\right)]\\ & \;\times \rho H_{U}-\mu H_{C}. \end{array}$$
